# Implementation of tuberculosis and cryptococcal meningitis rapid diagnostic tests amongst patients with advanced HIV at Kamuzu Central Hospital, Malawi, 2016–2017

**DOI:** 10.1186/s12879-022-07224-6

**Published:** 2022-03-05

**Authors:** Cecilia Kanyama, Maganizo B. Chagomerana, Chimwemwe Chawinga, Jonathan Ngoma, Idah Shumba, Wiza Kumwenda, Billio Armando, Tapiwa Kumwenda, Emily Kumwenda, Mina C. Hosseinipour

**Affiliations:** 1 The University of North Carolina Project-Malawi, Tidziwe Centre, Private Bag A-104, Lilongwe, Malawi; 2 Kamuzu Central Hospital, Ministry of Health, P.0 Box 149, Lilongwe, Malawi; 3 Partners In Hope, P.O Box 302, Lilongwe, Malawi; 4 Lighthouse Trust, Kamuzu Central Hospital, P.O Box 106, Lilongwe, Malawi

**Keywords:** Advanced HIV disease, Rapid diagnostic test, Cryptococcal disease, Tuberculosis, HIV, Malawi

## Abstract

**Background:**

Cryptococcal meningitis (CM) and tuberculosis (TB) remain leading causes of hospitalization and death amongst people living with HIV, particularly those with advanced HIV disease. In hospitalized patients, prompt diagnosis of these diseases may improve patient outcomes. The advanced HIV rapid diagnostic tests such as determine TB urine lipoarabinomannan lateral flow assay (urine LAM), urine X-pert MTB/RIF assay (urine X-pert), and serum/blood cryptococcal antigen test (serum CrAg) are recommended but frequently not available in many resource-limited settings. We describe our experience providing these tests in a routine hospital setting.

**Method:**

From 1 August 2016 to 31 January 2017, a prospective cohort study to diagnose TB and Cryptococcal meningitis using point of care tests was conducted in the medical wards at Kamuzu Central Hospital, in Lilongwe, Malawi. The tests offered were PIMA CD4 cell count, serum CrAg, urine LAM, and urine X-pert. The testing was integrated into an existing HIV/TB treatment room on the wards and performed close to admission time. Patients were followed until discharge or death in the ward.

**Results:**

We included 438 HIV-positive patients; 76% had a previously known HIV diagnosis (87% already on ART). We measured CD4 count in 365/438 (83%), serum CrAg in 301/438 (69%), urine LAM in 363/438 (83%), and urine X-pert in 292/438 (67%). The median CD4 count was 144 cells/ml (IQR 46–307). Serum CrAg positivity rate was 23 /301 (8%) and CM was confirmed by CSF Crag in 13/23 (56%). The majority of CM patients 9/13 (69%) started antifungal therapy within two days of diagnosis. Urine LAM and urine X-pert positivity rates were 81/363(22%) and (14/292 (5%) respectively. The positivity rate of urine LAM was higher in patients with low CD4 cell counts (< 100 cells/ml) and low BMI (< 18.5). Most patients with positive urine LAM started TB treatment on the same day. Despite the early diagnosis and treatment of TB and CM, the inpatient mortality was high; 30% and 25% respectively.

**Conclusion:**

Although advanced HIV rapid diagnostic tests are recommended, one key challenge in implementation is the limited trained personnel administering the tests. Despite the effective use of the point of care tests in the clinical care of hospitalized TB and CM patients, mortality among these patients remained unacceptably high. Henceforth we need to train other cadres apart from nurses, clinicians, and laboratory technicians to conduct the tests. There is an urgent need to identify and modify other risks of death from TB and CM.

*Trial registration:* Malawi National Health Science Research committee: Protocol # 1144. Registered 2 July 2014 and University Of North Carolina IRB #: UNCPM 21412, approved 13th October 2014.

## Introduction

Despite the successful roll-out of antiretroviral therapy (ART) programs in Sub-Saharan Africa, patients still present to hospital with advanced HIV disease (defined as a CD4 < 200/mm^3^ or WHO stage 3 or 4) and consequently, medical admissions and death due to HIV-related infectious diseases remain unacceptably high [1–5]. Mortality rates are high amongst people living with HIV (PLHIV) who are newly diagnosed with HIV, have not started ART, or have been on ART for less than a year. Most hospital deaths amongst PLHIV occur within two days of hospitalization [3, 6]. In Autopsy studies, major causes of death of hospitalized (inpatients)PLHIV are disseminated tuberculosis (TB) (37%) and cryptococcal meningitis (CM) (20%) [7–9]. Similarly, the causes of early mortality in outpatients with advanced HIV disease starting ART are TB (47%) and CM (12%) [10]. However, TB and CM remain undiagnosed at death in 38% and 50% of patients [10, 11].

A death audit conducted in 2004 at Kamuzu Central Hospital (KCH), a tertiary referral hospital in Lilongwe Malawi, reported a mortality rate of 24% amongst hospitalized PLHIV. A similar audit conducted eight years later at KCH reported the same mortality rate despite the marked expansion of ART nationally [3, 6]. In Blantyre (southern Malawi), despite a reduction in population-based HIV mortality and hospitalization, inpatient mortality for PLHIV remained constant from 2012 to 2019 at the tertiary referral hospital (Queen Elizabeth Central Hospital) [12].

The unwavering inpatient mortality trend in PLHIV can be addressed by identifying key health system failures [4]. One key health system failure is a delayed diagnosis of opportunistic infections in those with advanced HIV. The delayed diagnosis often leads to delays in offering rightful treatment. To improve the diagnosis of opportunistic infections and reduce inpatient mortality, KCH developed the following strategies: (1) restructuring wards to include a medical “short stay” unit where patients are stabilized and admitted and get routine opt-out HIV testing, using routine order sets for specified conditions [13], and (2) starting treatment for identified opportunistic infections and ART soon after diagnosis. Although these strategies are already in place, most hospital deaths in PLHIV still occur within two days of admission and usually before a diagnosis of HIV and opportunistic infection [2]. Therefore, there is still an urgent need for improved, rapid diagnostic testing on admission to the medical wards.

In areas with a high prevalence of TB and HIV, the addition of advanced HIV rapid diagnostic tests (ADHIV RDTs) for the diagnosis of TB and CM increases the yield of TB and CM. ADHIV RDTs also reduce mortality in some subgroups including those with CD4 < 100 cells/mm^3^, anemia, and those with suspected TB at admission [14]. Although urine LAM has the highest yield in those with a CD4 count below 100 cells/mm^3^, WHO guidance stipulates that urine LAM should be performed for all hospitalized patients regardless of CD4 count [15]. In outpatient clinics, CM screening using serum CrAg and preemptive treatment with antifungal drugs reduces mortality, is feasible, and can be cost effective [16, 17]. However, there is still a gap in the implementation and yield of routine screening for CM using serum CrAg in hospitalized HIV-positive patients.

While following the WHO recommendations for the management of advanced HIV infection, we adopted and assessed the impact of ADHIV RDTs in the medical wards at KCH for diagnosing TB and CM. We assessed an organizational model where we incorporated rapid diagnostic testing using urine LAM, urine X-pert, and serum CrAg into the general medical inpatient care setup at KCH, a tertiary level hospital in Malawi.

## Methods

### Study design and patients

KCH is a tertiary level hospital in the central region of Malawi and is a referral hospital for five other district hospitals. The hospital admits 8000 patients per year in the medical wards. Standard of care services at the time of the study included opt-out HIV testing and inpatient ART initiation, TB diagnosis through clinical and radiological criteria, microbiological fluid analysis (cell count, gram stain), sputum ZN and sputum X-pert MTB/RIF. There was no routine screening for CM. For suspected cases of CM, the diagnosis was made based on a suggestive history followed by confirmation by CSF analysis using Indian Ink stain.

We conducted a prospective cohort study where the ADHIV RDTS (PIMA CD4 cell count, urine LAM, urine X-pert, and serum CrAg) were introduced in the admission and inpatient medical care setup for 6 months—between 1 August 2016 and 31 January 2017. The TB/ HIV treatment room in the ward was modified to incorporate the required point of care test kits and testing algorithms.

All HIV-positive patients in the ward were eligible to have the ADHIV RDTs. However, because of limitations in the availability of test kits and no personnel to perform the tests on weekends and during the night, not all HIV-positive patients had the ADHIV RDTs. The ADHIV RDTs supplemented the routine care that patients received. The PIMA CD4 cell count and urine LAM were done in the ward while urine x-pert, serum, and CSF CrAg were done at the laboratory. All Patients with positive serum CrAg had a confirmatory lumbar puncture (LP) for CM. Patients already on TB treatment did not have a urine LAM or urine x-pert test but were screened for CM.

We obtained ethical approval from the Malawi National Health Research Committee and University of North Carolina at Chapel Hill (UNC) IRB. Written consent was required because these tests were not part of routine care.

### Protocol training

Immediately before the start of the study, we conducted training for the study clinician and nurses. The study principal investigator and microbiology department technician at the UNC project led the training. The study training included a theory segment and a practical segment on collecting samples and performing the urine LAM, serum CrAg, and PIMA CD4 cell test. Urine x-pert test was done at UNC project Laboratory and was not part of the training sessions. Using the trainer of trainers’ model, the training propagated to all medical admissions and inpatient care teams including nurses and clinicians. The study nurse and clinician continued to supervise all staff during the period.

### Procedures

The study nurse identified all recently admitted PLHIV in the ward during working hours and as close to the admission time as possible. Once identified, eligible patients or their guardians provided informed consent for study participation.

After enrollment, a urine specimen was collected in a sterile container and tested in the ward for urine LAM and transferred to UNC Project laboratory for urine X-pert testing. Urine LAM was defined as positive using the grade 1 cutoff on the manufacturer’s post-2014 reference card. CD4 cell counts were done by Alere PIMA® CD4 machine. The study nurse, study clinician, or the hospital clinicians/nurses performed these tests. The urine LAM test result and CD4 were available to the patient within an hour of sample collection. The UNC-Project laboratory also performed serum and CSF CrAg. The goal was to have all results within 24 h of sample collection. All tests were performed according to the manufacturer’s instructions. As part of routine clinical care procedures at the hospital, all TB suspects who could submit sputum received immediate sputum X-pert MTB/RIF tests per the proposed Malawi algorithm. Other routine care procedures i.e. microbiological fluid analysis, sputum Ziehl–Neelsen stain, X-rays, sonography, were requested by the attending clinicians as needed.

We documented all results in the patient’s files for review by the attending clinician in the ward. The clinical management and provision of TB and or CM treatment were at the discretion of the attending clinician by local practice and national guidelines. TB diagnosis was either confirmed (positive urine LAM/x-pert, Sputum AAFB/x-pert MTB/RIF) or presumptive (based on clinical presentation and imaging only). Using a standardized case report form, the study team collected information from the admission forms, ADHIV RDTs logs, and clinical notes on demographics, final diagnosis, and treatment outcomes for patients treated for TB and CM.

### Statistical analysis

The data collected was double entered into a password-protected customized Microsoft access 2013 Database. Stata version 14.2 was used for all statistical analyses.

Categorical variables were summarized as counts and percentages. Continuous variables were summarized using medians and their corresponding interquartile ranges (IQRs). We assessed the association between baseline characteristics and HIV diagnosis status at admission using fisher’s exact test (categorical variables) and Wilcoxon rank-sum test (continuous variables). The Fisher’s exact test was also used to assess the association between CD4 count and positivity of each of the following: Urine LAM, urine X-pert, and serum CRAG. We also used the Fisher exact test to compare Urine LAM positivity across different BMI levels. We calculated the sensitivity and specificity of urine LAM with urine X-pert as the gold standard. Kappa statistic was used to compare the agreement of test results between urine LAM and urine X-pert.

## Results

The ADHIV RDTs –PIMA CD4 cell count, urine LAM, urine x-pert, serum CrAg were successfully incorporated into the medical admissions and inpatient care. During the intervention period, 3069 patients were admitted to the medical wards. We could only ascertain HIV status in 2165 (70%) of the admissions. The HIV-positivity rate of this group was 980/2165 (45%). 87% (849/980) were known as HIV positive and were already on ART at this hospital admission.

Of the 2165 PLHIV ascertained, only 438 PLHIV were enrolled in the study. Among the enrolled patients, 335/438 (76%) were already known as HIV-positive at admission, 73/438 (17%) were newly diagnosed with HIV infection during admission and the time of HIV diagnosis could not be ascertained in 30/438 (7%). The baseline characteristics of the enrolled patients were similar among participants with known, new, and unknown diagnoses except for gender (p = 0.01) and CD4 cell count (p = 0.02) (Table 1).

**Table 1:** Characteristics of PLHIV who were screened for tuberculosis and cryptococcal meningitis while they were admitted to the medical wards at KCH, Lilongwe, Malawi, 2016–2017

Characteristic	Total N (%)	HIV diagnosis on admission			P-value*
		Old diagnosis N (%)	New diagnosis N (%)	Unknown^*^ N (%)	
Gender					
Female	205 (47.8)	164 (50.2)	24 (32.9)	17 (58.6)	0.01
Male	224 (52.2)	163 (49.8)	49 (67.1)	12 (41.4)	
Age (years)					
14–20	15 (3.8)	11 (3.6)	4 (5.7)	0 (0.0)	
21–30	87 (21.7)	65 (21.1)	16 (22.9)	6 (27.3)	
31–40	163 (40.8)	124 (40.3)	29 (41.4)	10 (45.4)	0.92
41–50	85 (21.2)	67 (21.7)	13 (18.6)	5 (22.7)	
50 +	50 (12.5)	41 (13.3)	8 (11.4)	1 (4.6)	
BMI					
< 18.5	80 (27.7)	64 (28.2)	13 (27.1)	3 (21.4)	
18.5–24.9	185 (64.0)	141 (62.1)	33 (68.7)	11 (78.6)	0.79
25.0–29.9	18 (6.2)	17 (7.5)	1 (2.1)	0 (0.0)	
30 +	3 (2.1)	5 (2.2)	1 (2.1)	0 (0.0)	
CD4 count					
≤ 100 cells/mm^3^	152 (41.5)	119 (41.9)	20 (32.3)	12 (63.2)	
101–200 cells/mm^3^	69 (18.9)	46 (16.2)	20 (32.3)	3 (15.8)	0.02
≥ 200 cells/mm^3^	145 (39.6)	119 (41.9)	22 (35.4)	4 (21.0)	
WHO clinical stage					
Stage 1	42 (12.5)	30 (11.4)	11 (19.6)	1 (6.7)	
Stage 2	11 (3.3)	10 (3.8)	1 (1.8)	0 (0)	0.08
Stage 3	215 (64.0)	164 (62.1)	40 (71.4)	10 (66.7)	
Stage 4	68 (20.2)	60 (22.7)	4 (7.2)	4 (26.6)	

9 missing gender, 38 missing age, 149 missing BMI, 9 missing gender, 73 missing CD4 cell count, and 103 missing WHO clinical stage

*Fisher’s exact test

We measured CD4 cell count in 365/438 (83%), serum CrAg in 301/438(69%), urine x-pert in 292/438 (67%) and urine LAM in 363/438 (83%) of PLHIV. The median CD4 count was 144 (IQR: 46–307). The overall positivity rate for serum CrAg was 7.6% (23/301). Serum CrAg positive test was higher in patients with CD4 < 100 cells/ml (11.5%) compared to patients with CD4 101–200 cells/ml (5%) and CD4 > 200 cells/ml (4%), p = 0.04 (Table 2). Of the 23 patients who tested positive for serum CrAg,13 (56%) were confirmed CM by CSF CrAg; 9/13 (69%) of CM started antifungal therapy within two days of LP while 4/13(31%) died shortly after admission before treatment could be administered.

**Table 2:** Results of rapid diagnostic tests for PLHIV admitted in the medical wards and screened for TB and CM stratified by the CD4 cell count at KCH, Lilongwe, Malawi, 2016–2017

Characteristic	Total N (%)	CD4 cell count			P-value^*^
		≤ 100 cells/mm^3^ N (%)	101–200 cells/mm^3^ N (%)	> 200 cells/mm^3^ N (%)	
Serum CrAg					
Negative	278 (92.4)	108 (88.5)	52 (91.2)	118 (96.7)	
Positive	23 (7.6)	14 (11.5)	5 (8.8)	4 (3.3)	0.04
Urine LAM					
Negative	282 (77.7)	103 (68.2)	55 (80.9)	124 (86.1)	
Positive	81 (22.3)	48 (31.8)	13 (19.1)	20 (13.9)	0.001
Urine X-pert					
Negative	278 (95.2)	104 (88.9)	57 (98.3)	117 (100)	
Positive	14 (4.8)	13 (11.1)	1 (1.7)	0 (0)	< 0.001

Among those with CD4 cell count results, 64 did not have CrAg or CrAg results were missing; 2 did not have urine LAM results, and 73 did not have urine X-pert test or urine X-pert results were missing

^*^Fisher’s exact test

Urine LAM positivity rate was 22% overall (81/363) and significantly higher in patients with CD4 < 100 cells/ml (31.8%) compared to patients with CD4 101–200 cells/ml (19.1%) and CD4 > 200 cells/ml (13.9%), p = 0.001 (Table 2). Positive Urine LAM was also significantly higher in patients with a low BMI than normal/overweight (25/80 (32%) and 35/207 (17%) respectively (p < 0.005) and WHO stage 3 and 4 (p < 0.001).

Among 334 patients who had both urine LAM and urine X-pert testing, the number of TB cases detected using urine LAM [76 (22.8%)] was higher compared to the number of TB cases detected using urine X-pert [16 (4.8%)], proportion difference = 0.18; 95% CI: 0.13– 0.23 (p < 0.001) Of the 16 patients who tested positive using urine X-pert, 13 tested positive using urine LAM (LAM sensitivity = 81.3%; 95% CI: 54.4, 96.0%). The agreement in test results between Urine X-pert and Urine LAM was 80.2% and kappa statistic for agreement was 0.22, 95% CI: 0.11- 0.33. (Table 3).

**Table 3:** Performance of urine x-pert and Urine LAM for PLHIV admitted in the medical wards and screened for TB at KCH, Lilongwe, Malawi, 2016–2017 N = 334

		Urine X-pert		
		Positive N (%)	Negative N (%)	Total N (%)
Urine LAM	Positive	13 (81.3)	63 (19.8)	76 (22.7)
	Negative	3 (18.7)	255 (80.2)	258 (77.3)
			Estimate (95% CI)	
	Sensitivity		81.3% (54.4, 96.0)	
	Specificity		80.2% (75.4, 84.4)	
	Positive predictive value		17.1% (9.4, 27.5)	
	Negative predictive value		98.8% (96.6, 99.8)	
	Agreement		80.2% (75.6, 83.4)	
	Kappa statistic		0.22 (0.11, 0.33)	

Overall, 97 patients were diagnosed with confirmed TB (84%) or presumed TB (16%).

Because of the poor inpatient record-keeping system, only 241 (55%) patients had a known outcome after hospitalization. Of these, 46 /241 died (mortality rate = 19.1; 95% CI: 14.6–24.6). Among all the patients diagnosed with TB (97), 61 had known hospitalization outcomes. Of the 61 TB patients with known hospitalization outcomes, 18 patients died (mortality rate = 29.5%; 95% CI: 19.2–42.4) despite the prompt initiation of TB therapy within 24 h of diagnosis.

Only 16 files of the 23 files for patients who had serum CrAg positive were available to ascertain hospitalization outcomes. Of these 16 patients, 4 died (mortality rate = 25.0%; 95% CI: 8.6–54.3), had confirmed CM and died before treatment.

## Discussion

The model of integrating HIV and TB treatment has been successful in outpatient clinics treating HIV patients co-infected with TB [18, 19]. We now demonstrate the successful use of ADHIV RDTs in hospital services at a tertiary level hospital in Malawi as a preface to scale up in the country. The program was successful because of didactic training, practical demonstrations of use, the ease of testing using a preset algorithm, and continuous supervision of the new intervention. In addition, the ADHIV RDTs were incorporated into the existing system already providing ART and TB drugs in the medical wards. This helped in reducing the time between diagnosis and treatment of HIV, TB, and CM.

Not surprisingly, CrAg positivity was higher in the hospital setting than in previously documented outpatient settings in Malawi.The prevalence of CrAg positivity among outpatients with CD4 < 100 cells/ml and WHO stage IV was 3.5% (95% CI: 0–8.4%) and 5.0% (95% CI: 0–15%), respectively [17]. A meta-analysis shows in concordance to our setting that the prevalence of cryptoccaemia is higher in hospitalized patients (9.8% [95% CI, 4.0–15.5%]) compared with outpatients (6.3% [95% CI, 5.3–7.4%]) [20]. In our study, a higher inpatient CrAg positivity rate may have been a result of screening all PLHIV hospitalized regardless of CD4 count or WHO stage. Our finding that more than half of the serum CrAg positive patients had confirmed CM supports the need for CrAg screening and preemptive antifungal treatment at the time of HIV diagnosis for all patients with advanced HIV disease [8, 15].

The urine X-pert test did not significantly add to the detection of disseminated TB beyond urine LAM. This is also well supported in a clinical trial which showed that urine LAM had major incremental diagnostic benefit with urine X-pert contributing few additional diagnoses [14]. The Urine LAM is also easier to use in our setting as it was conducted on the ward whereas the urine X-pert required transportation and testing in the laboratory. The lower cost, easier use, and higher performance of the Urine LAM make it a logical test for investment and incorporation to increase TB diagnosis. Urine X-pert has no significant addition to urine LAM as a rapid diagnostic test for TB and therefore can be eliminated from advanced HIV care algorithms for resource-limited settings.

Lower CD4 count (≤ 200 cells/mm^3^) was the most important predictor of a positive Urine LAM and serum CrAg test. Hence, CD4 count remains an important marker of the performance of the ADHIV RDTs. However, considering the time and cost of the CD4 cell count test, we need to evaluate the cost-effectiveness of adding a CD4 cell count test in the hospitalized patient as a benchmark for performing ADHIV RDTs. Certainly, the outpatient models show the cost-effectiveness of preemptive screening for cryptococcal disease regardless of CD4 [14, 19]. In our study, half of the patients had CD4 counts below 200 cells/mm^3^ but positive cases of LAM and CrAg were also found among patients with CD4 > 200 cells/mm^3^ emphasizing the need to follow advanced HIV disease full package model for all hospitalized patients [15].

Despite the substantial coverage of ART in Malawi (86%) [22], hospitalization for HIV related complications remains high (45%). In this study, inpatient mortality was 30% for those with TB and 21% within two weeks of CM diagnosis, similar to r previously reported clinical trials [23]. There is some evidence of long-term impact on mortality using ADHI RDTs for TB but not for CM [14, 21, 24]. Our study was not able to conduct routine HIV RNA to determine the rate of treatment failure among these inpatients with ART experience or fully evaluate their adherence. However, the treatment failures identified in hospital admissions represent failures in the outpatient systems of the HIV program. These system problems may include inadequate pre-ART screening for opportunistic infections, missed treatment failure evaluations, weak retention strategies for defaulters, late diagnosis of HIV infection, and late presentation for acute illnesses. The short time from admission to death for our cases highlights that real-time inpatient diagnosis may be too late to significantly alter mortality for our inpatient population. Strengthening outpatient programs is needed to reduce mortality.

We could not assess all inpatients due to high volumes and limited resources. However, we believe the patients in this study reflect a true picture of the situation in the medical department. We were also unable to ascertain all inpatient outcomes that may have affected our overall inpatient mortality estimate. The study staff looked through all discharge/death folders and notes available in the wards but some files were missed because of inadequate record keeping. The hospital record-keeping is poor because the hospital uses paper-based unstandardized documentation. Most missing files would presumably be of patients who died as death files are taken away from the wards expeditiously to perform death audits. However, TB and CM diagnoses and mortality estimates in this study align with previous work in Malawi [3, 6, 12, 23].

Key challenges in the implementation of ADHIV RDTs noted were inability to assess all admissions with existing staff, high turnover of staff requiring continuous mentorship, renovation of space to include adequate lighting for visualization of ADHIV RDTs results, and the time required to perform a test that required dedicated staff. These thematic areas need further exploration as the scale-up ADHIV RDTs for management of opportunistic infections continues.

## Conclusion

ADHIVRDTs for TB and CM in hospitalized HIV-positive patients can lead to improvements in the timely diagnosis and treatment of TB and CM. The urine LAM test remains easy to perform assay and has the best yield for disseminated TB. One implementation challenge is limited trained personnel to administer the tests. Therefore, it is necessary to train other cadres apart from nurses, clinicians, and laboratory technicians to conduct these tests. Despite the rapid diagnosis and initiation of treatment, mortality remains high for TB and CM, suggesting the need for earlier diagnosis in an outpatient setting. Research is imperative to define risks for high mortality in patients with advanced HIV disease to improve outcomes.

## Data Availability

The full data set supporting the conclusions of this article is held by the corresponding author and can be made available on request. Email: ckanyma@med.unc.edu.

## Abbreviations

ART: Antiretroviral therapy
CM: Cryptococcal meningitis
Crag: Cryptococcal antigen
HIV: Human immunodeficiency virus
KCH: Kamuzu Central Hospital
LAM: Lipoarabinomannan
ADHIV RDTS: Advanced HIV Rapid diagnostic tests
TB: Tuberculosis
PLHIV: People living with HIV
LP: Lumbar puncture
UNC: University of North Carolina
